# Tuberculosis Case Finding in Kulon Progo District, Yogyakarta, Indonesia: Passive versus Active Case Finding Using Mobile Chest X-ray

**DOI:** 10.3390/tropicalmed9040075

**Published:** 2024-04-04

**Authors:** John Silwanus Kaku, Riris Andono Ahmad, Stephanie Main, Dwi Oktofiana, Bintari Dwihardiani, Rina Triasih, Philipp du Cros, Geoffrey Chan

**Affiliations:** 1 Center for Tropical Medicine, Faculty of Medicine, Public Health and Nursing, Universitas Gadjah Mada, Yogyakarta 55281, Indonesia; 2 Department of Biostatistics, Epidemiology and Population Health, Faculty of Medicine, Public Health and Nursing, Universitas Gadjah Mada, Yogyakarta 55281, Indonesia; 3 International Development, Burnet Institute, Melbourne, VIC 3000, Australia; 4 Kulon Progo District Health Office, Yogyakarta 55611, Indonesia; 5 Department of Pediatric, Faculty of Medicine, Public Health and Nursing, Universitas Gadjah Mada/Dr. Sardjito Hospital, Yogyakarta 55281, Indonesia

**Keywords:** Tuberculosis, active-case finding, mobile chest X-ray

## Abstract

Active-case finding (ACF) using chest X-ray is an essential method of finding and diagnosing Tuberculosis (TB) cases that may be missed in Indonesia’s routine TB case finding. This study compares active and passive TB case-finding strategies. A retrospective study of TB case notification was conducted. Data between 1 January and 31 December 2021, was used. The population in this study were TB cases notified from Kulon Progo District health facilities, including those found through routine activities or active-case findings. A total of 249 TB cases were diagnosed in Kulon Progo in 2021, and 102 (41%) were bacteriologically confirmed. The TB patients’ ages ranged from 0 to 85 years (median 52, IQR 31–61). The majority of cases were male (59%, 147/249) and mostly among people aged 15–59 (61.4%, 153/249). The proportion of clinical TB diagnoses among cases found from active-case findings was 74.7% (68/91) while the proportion among passive-case findings was 50% (79/158). Active-case finding contributed 91 (36.5%) TB cases to the total cases detected in Kulon Progo in 2021. The use of chest X-rays in active-case findings likely contributed to the detection of a higher proportion of clinical TB than in passive-case findings.

## 1. Introduction

In recent years, Tuberculosis (TB) has been the world’s leading cause of death from a single infectious agent worldwide surpassed only in 2022 by COVID-19 [1]. In 2021, the World Health Organization (WHO) estimated that around 10.6 million people worldwide fell sick with TB, a 4.5% increase from 2020 [2]. Indonesia has the second-highest global TB caseload, with a TB incidence in 2020 of 301 per 100,000 population, and a mortality rate of 34 per 100 thousand people [3].

One essential pillar of the WHO’s End TB strategy is early diagnosis and treatment [4]. Unfortunately, many countries rely on passive case finding, in which only those who seek care at health facilities are examined for TB [5,6]. As a result, many cases are missed, particularly in people who are asymptomatic, have mild symptoms, or do not seek treatment at health facilities. This can lead to continuing transmission, worsening of the patient’s condition, and even death [6].

Active case finding (ACF) is an important method of finding and diagnosing TB cases that may be missed by passive case finding. ACF involves systematic screening for active TB outside health facilities in high-risk populations, such as those living in congregate settings [6]. Several studies have shown that ACF can increase the number of reported cases and reduce the prevalence of TB in the community [7]. However, a recent systematic review found inconsistent results and low-quality evidence for the benefit of active case finding on incidence and prevalence. For example, randomised control trials (RCT) utilising community mobilisation and sputum drop-off in South Africa did not affect TB prevalence, whereas an RCT in Vietnam utilising door-to-door sputum testing for everyone significantly reduced TB prevalence [8]. Nevertheless, the review concluded that ACF might effectively change TB epidemiology if it is designed for specific settings and delivered with high coverage. However, this highlights the need for further evaluation of ACF programs in different settings incorporating alternate delivery methods and diagnostic strategies [8].

ACF activities are highly recommended in high burden countries such as Indonesia and may help to reduce the underdiagnosis of TB [5]. The WHO consolidated guidelines on systematic screening also recommend that systematic screening should be conducted among household contacts of TB patients, people living with HIV, and the general population in areas with an estimated TB prevalence of 0.5% or higher [9]. In Indonesia, there are gaps in case detection and reporting: a TB inventory study in Indonesia in 2017 found that 44% of TB cases had not been reported, and 30.4% had not been detected [10]. Case detection has been further impacted by the COVID-19 pandemic, resulting in fewer cases reported [2]. Comprehensive TB services incorporating ACF—including symptom screening and diagnostic testing—have not been widely implemented, described, or evaluated in Indonesia [11].

Zero TB Yogyakarta (ZTBY) is a collaborative project between the Center for Tropical Medicine Universitas Gadjah Mada, the health offices of Yogyakarta Province, Kulon Progo District and Yogyakarta City, and the Burnet Institute, Melbourne. The ZTBY project conducted TB ACF in Kulon Progo Regency and Yogyakarta City in 2020 and expanded to additional districts in 2022. ZTBY active case finding screens for TB among target groups using symptom screening and mobile chest X-ray (a vehicle equipped with an X-ray device), with microbiological testing for those who screen positive. The groups targeted by the ACF have included residents of low socio-economic areas, household contacts of TB patients, people living with HIV (PLHIV), residents in prisons, residents of orphanages, Islamic boarding schools, healthcare workers, and those living in areas with high TB cases [12]. To date, the impact of the ACF service on local case notifications and populations reached has not been evaluated. Such an evaluation could help to understand which case-finding strategies are most effective in detecting TB cases and what needs to be executed in order to improve the current TB management systems in the region.

This study compares TB cases found through passive and active-case findings in the Kulon Progo District in 2021. The aims of this research were to: describe the proportion of notified TB cases that were diagnosed by active case finding; to investigate whether demographic and clinical factors differed between active-case finding and passive-case finding; to assess the extent of underreporting of TB cases; and to describe the characteristics of TB cases that were not reported.

## 2. Materials and Methods

This study is a retrospective review of TB case notification data. The population in this study were TB cases notified in Kulon Progo District between 1 January and 31 December 2021. It includes persons with TB found through routine case finding and those found from active-case finding activities implemented by the Zero TB Yogyakarta program. 

### 2.1. Setting

**Figure 1:**
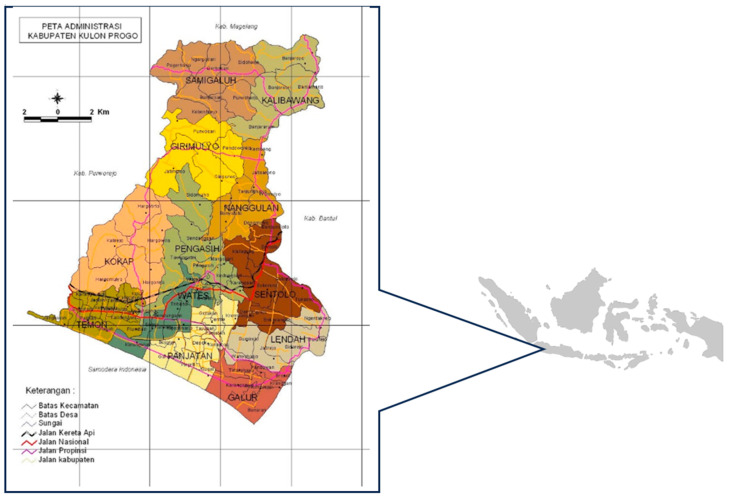
Geographical map of Kulon Progo District, Yogyakarta Province, Indonesia. Source: https://peta-kota.blogspot.com (accessed on 10 February 2023).

Kulon Progo District is a rural district in Yogyakarta Province, with an area of 586.28 km^2^_._ The total population is 443,283 people spread over 12 sub-districts, 87 villages, and 917 sub-villages [13] (Figure 1). Of the total population, 49% are men, and 51% are women. The proportion of the working-age population (15–64 years) is 67.8%. There are 21 primary healthcare centres (puskesmas), two government hospitals, and seven private hospitals that provide TB services. There are two puskesmas (Kalibawang and Galur 2) and two hospitals (RSUD Wates and RSUD Nyi Ageng Serang) that can carry out TB microbiological examinations using the GeneXpert platform with Xpert MTB/RIF cartridges [14].

Prior to the commencement of Zero TB Yogyakarta activities in one subdistrict of Kulon Progo in 2020, TB case findings in Kulon Progo usually consisted of passive case findings in which TB cases were detected among persons seeking care at the health facilities. Additionally, household contacts of TB patients (index cases) were investigated by primary health centres in collaboration with trained local village cadres. Sputum samples were collected from household contacts and tested. Kulon Progo notified 314 cases in 2017, 252 in 2018, and 304 in 2019. There were 163 bacteriologically confirmed pulmonary TB cases notified from Kulon Progo in 2017, 127 in 2018, and 128 in 2019.

Active case finding in this study is a part of Zero TB Yogyakarta activities that involved mobile chest X-rays for TB screenings. Zero TB Yogyakarta’s ACF was conducted in sub-villages where TB cases had been found in the last five years. We also targeted groups for screening such as household contacts of TB patients, residents at Islamic boarding schools, orphanages, and people living with HIV. We held multi-sectoral meetings with puskesmas, local governments, cadres, and community leaders two weeks before conducting an ACF in a locale. These meetings were used to explain the ACF activity to key stakeholders and discuss their concerns and potential challenges in conducting ACF. These stakeholders were then asked to invite the target population.

**Figure 2:**
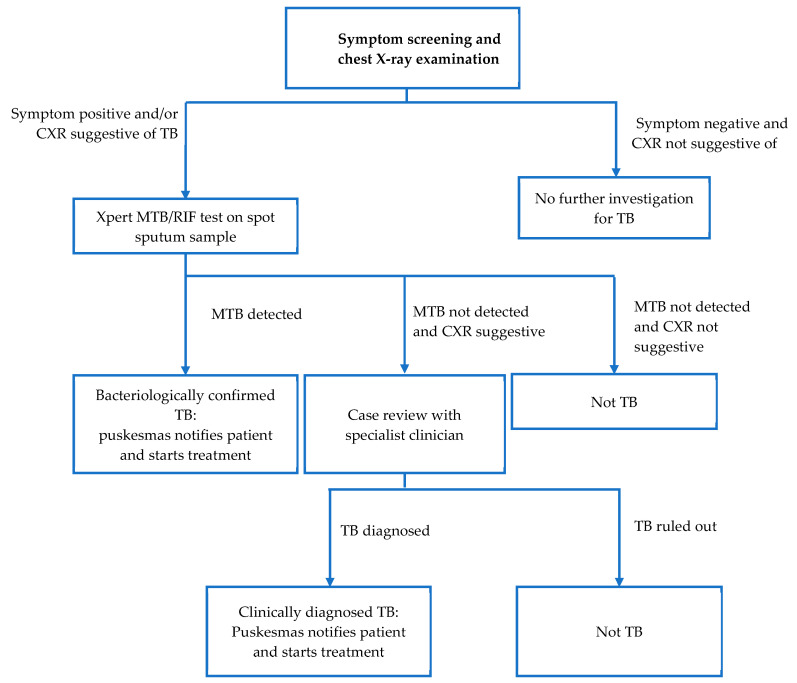
The ACF screening algorithm.

The ACF service was set up at sites within or proximal to the target village. The specific site for setting up the mobile ACF service was chosen based on its accessibility to the target population. People presenting to the ACF service were registered, checked for TB symptoms, and had a chest X-ray examination performed. From January to April 2021, the X-ray results were read by a doctor within the ACF team. In May 2021, the ACF service commenced using qXR software (qure.AI, India) for computer-assisted X-ray interpretation. Participants with TB symptoms or a chest X-ray suggestive of TB (based on the doctor’s interpretation or a qXR score > 0.5) were asked to produce a single spot sputum sample on site. Spot sputum samples were then transported to a laboratory for testing with GeneXpert MTB/RIF test. The puskesmas whose catchment covered the target village then entered the data of persons with presumptive TB in the SITB (Sistem Informasi Tuberkulosis—Indonesia’s national case-based electronic TB surveillance system). The laboratory performing the test was then expected to update the SITB with the test results. If the GeneXpert test results were positive, the puskesmas informed the patient. If the GeneXpert result was negative but the chest X-ray (CXR) was suggestive of TB, the puskesmas doctor in coordination with the ACF team consulted with clinician specialists to diagnose or rule out clinical TB. All patients with TB diagnosis, both bacteriological and clinical, were invited to start treatment at their nearest puskesmas. Figure 2 shows the screening and diagnostic algorithm for Zero TB Yogyakarta’s ACF. Data on screening, diagnosis, and treatment of patients screened by the ZTBY ACF service was recorded in a ZTB REDCap database. Data on persons with presumptive TB screened by ZTBY ACF was provided to the puskesmas for entry into SITB; to which the puskesmas would then add data on TB diagnosis and treatment.

**Figure 3:**
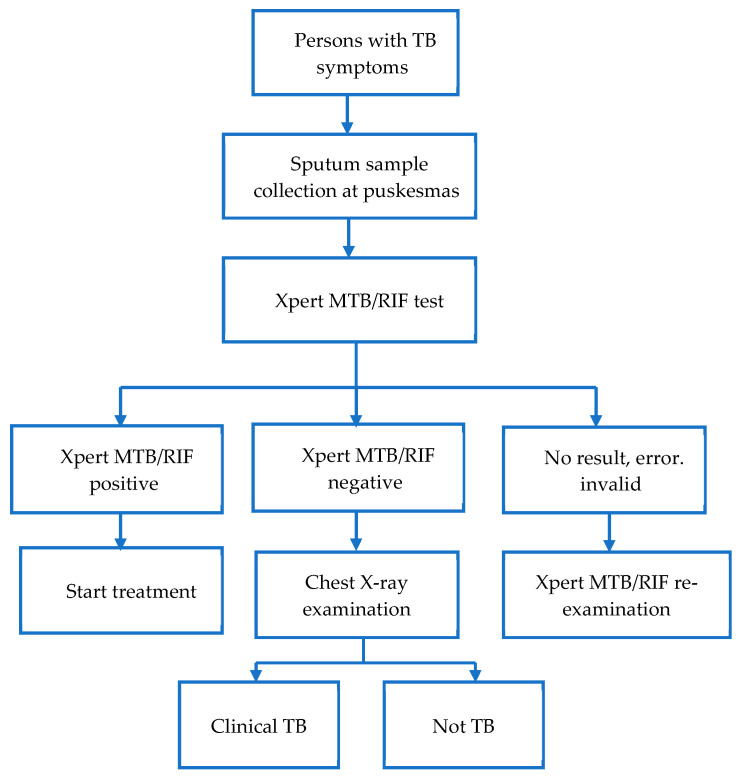
The PCF screening algorithm.

In passive-case findings, patients presenting to the puskesmas with TB symptoms were investigated for TB. These patients were asked to produce an early morning sputum sample to be tested with a GeneXpert MTB/RIF test. The patients were recorded in SITB once the sputum samples were collected and transported to the laboratory. If the test result was positive, the puskesmas invited the patient to start treatment. If the result was negative, the puskesmas referred the patient for a chest X-ray examination and used the chest X-ray to help decide if the patient had clinical TB or not. The puskesmas were responsible for reporting on the diagnosis and treatment initiation of bacteriological and clinical cases in the SITB. Figure 3 shows the PCF algorithm.

### 2.2. Data Collection and Analysis

This study utilised secondary data collected in 2021 from the SITB in Kulon Progo and Zero TB Yogyakarta’s REDCap database. The SITB is the Indonesian Ministry of Health’s case-based digital TB surveillance system. Health facilities such as hospitals, puskesmas, clinics, laboratories, and pharmacy installations, are required to use the platform to manage and report TB cases in Indonesia. REDCap (Research Electronic Data Capture) is a secure, web-based software platform that supports data capture for research studies [15,16].

Zero TB Yogyakarta collected demographic and clinical data on persons presenting to its ACF services using REDCap electronic data capture tools on a server hosted by the Universitas Gadjah Mada. The clinical data included screening data, microbiological investigations, radiological investigations, TB diagnosis, TB treatment initiation, and TB treatment outcomes.

Persons undergoing microbiological investigation and starting TB treatment are meant to be entered into the SITB, regardless of whether they were initially screened from passive or active case findings. Persons initially screened through passive-case findings were recorded and reported in the SITB from puskesmas registers. For persons screened by Zero TB Yogyakarta ACF, data of persons who had a positive screening result (presumptive TB) were regularly exported from the REDCap database and provided to the catchment puskesmas for entry into the SITB. This exported data included personal details, symptom screening results, details of chest X-ray examination, and details of sputum collection. GeneXpert results from presumptive TB cases seen at Zero TB Yogyakarta’s active-case findings were followed up by Zero TB Yogyakarta and provided to the puskesmas to update the SITB records. The puskesmas was then responsible for entering details of TB diagnosis and TB treatment in SITB. Based on the puskesmas data, Zero TB Yogyakarta updated the REDCap database with details of diagnosis and treatment.

Two data sets were used for this study. The first consisted of SITB case-level data of patients recorded by puskesmas in Kulon Progo during the study period. The second data set consisted of Zero TB Yogyakarta REDCap data on persons screened by the Zero TB Yogyakarta ACF service in Kulon Progo. A national identification number (Nomor Induk Kependudukan/NIK) is recorded in the SITB and in the ZTBY REDCap data. This national ID number was used to link the ZTBY REDCap data with the SITB data. After linkage, identifiable data were removed from the dataset and the data were analysed using Stata version 17. Patients were considered to have been found through active-case finding if they were in the Zero TB Yogyakarta REDCap data. Patients were considered to be found through active-case finding and notified in SITB if the national ID number in SITB matched an ID number in Zero TB Yogyakarta REDCap. Patients were considered as found through passive-case finding if they were notified in the SITB and were not matched with a national ID number in REDCap. Categorical variables were analysed by frequency and proportion.

### 2.3. Ethical Approval

Ethics approval was obtained from The Medical and Health Research Ethics Committee (MHREC), Faculty of Medicine, Public Health and Nursing Universitas Gadjah Mada.

## 3. Results

Between 1 January and 31 December 2021, a total of 249 TB cases were diagnosed in Kulon Progo, of which 102 (41%) were bacteriologically confirmed (Table 1). The TB patients’ ages ranged from 0 to 85 years (median 52, IQR 31–61) (Table 1). Most cases were people aged 15–59 (61.4%, 153/249) and male (59%, 147/249) (Table 1). The proportion of clinical TB diagnoses among cases found from active-case findings was 74.7% (68/91) while the proportion among passive-case findings was 50% (79/158) (Table 1). Active-case finding contributed 91 (36.5%) TB cases to the total cases detected in Kulon Progo in 2021 (Table 1).

Zero TB Yogyakarta’s ACF screened 12,406 participants in 2021 and 12,019 (96.8%, 12,019/12,406) of them underwent chest X-rays. Those with symptoms or a chest X-ray result suggestive of TB (12.2%, 1514/12,406) had their sputum taken for Xpert MTB/RIF rapid molecular diagnostic testing. There were 23 (0.18%, 23/12,406) ACF participants who had a bacteriologically confirmed diagnosis of TB on GeneXpert testing, and there were 68 (0.54%, 68/12,406) ACF participants diagnosed with TB clinically. 

There were 36 cases from active case finding that were not found in the SITB data. Of these, 27 (75%) were recorded in the REDCap database as having started TB treatment. Of these 36 cases, 13 (36.1%) had a treatment start date outside the period for which data from the SITB was exported. The remaining 23 (63.9%) were categorised as not reported. Most of the unreported cases from ACF were clinically diagnosed with TB (86%, 20/23) and in univariate analysis, clinically diagnosed cases had 5.57 times higher odds of being unreported (95% CI 1.84–24.2, *p* = 0.007). Table 2 shows the univariate analysis of risk factors for non-reporting.

## 4. Discussion

In this study examining 2021 TB notification data from Kulon Progo district in Indonesia, three key findings were made: (1) active-case findings contributed a high proportion of the total number of TB cases detected in the district; (2) active-case finding detected more clinical TB than bacteriological compared to passive case finding; and (3) 46% of cases diagnosed through active-case finding were not reported in the SITB, the national TB reporting database in 2021.

ACF contributed to 35.7% of the 249 cases detected in Kulon Progo district in 2021. In comparison, 239 cases were notified in the preceding year (2020) but still below the pre-COVID notifications in 2017, 2018, and 2019. Several studies have shown increased TB case detection through ACF [17,18,19,20]. For example, community-based ACFs in India and Cambodia have been reported to increase TB case detection beyond what is found through routine case-finding activities [16,18]. Our study design does not allow us to infer the extent to which the 91 cases found through ACF represent additional yield over routine case finding in Kulon Progo and these persons might still have presented to health facilities in the absence of ACF. Importantly, during COVID-19, the impact of the pandemic may have influenced care-seeking and care-provision for TB diagnosis. Although Kulon Progo reported fewer notifications in 2020 and 2021 than in preceding years, it is nonetheless possible that the implementation of ACF mitigated reductions in TB case findings due to COVID-19. Despite this uncertainty, the yield of cases from ACF suggests it is acceptable and feasible in Kulon Progo, and therefore, could be viable as an approach for finding cases among populations that might not promptly seek care for TB at health facilities.

Notably, among ACF cases, there were higher proportions of cases in people aged over 60 years, of males, and of persons who were employed. These are perhaps indications that ACF is more successful than PCF in reaching and finding cases among certain population groups. The study period coincided with the COVID-19 pandemic and it is, therefore, also possible that the observed differences between ACF and PCF cases reflect how case-finding strategies were differentially affected by the pandemic and its impact on care seeking for TB.

The proportion of clinically diagnosed TB among cases detected from ACF was higher than the proportion among those detected from passive case finding. This finding could be due to differences in the target population, as well as differences in the screening algorithm. The high proportion of clinically diagnosed TB from ACF in our study is consistent with findings from other studies, including a study by Saunders et al. based on 10 years of data on ACF in Peru [21,22,23].

The use of chest X-ray screening in the ACF model of care is an important point of difference with PCF in Kulon Progo. Chest X-ray services are not available at most of the puskesmas in Kulon Progo and hence are not routinely used in PCF. Chest X-ray is important in TB screening and diagnosis and its use can result in increased case detection relative to case finding without chest X-ray [24]. In Indonesia, chest X-ray has not been widely used for TB ACF and case findings have focused on bacteriologically confirmed cases. Hence, the difference in the proportion of clinical cases between the two case-finding methods could reflect that ACF is finding cases that would be missed without a CXR examination.

The higher proportion of clinical TB diagnoses among cases detected from ACF could also be influenced by differences between the target population for ACF compared to PCF. Notably, whereas PCF relies on people who are already unwell to present to a facility for diagnosis, the community-based screening of high-risk populations by ZTBY ACF was expressly aimed at trying to detect TB earlier. Hence, individuals screened at ACF may be more likely to be asymptomatic and to test negative on GeneXpert MTB/RIF compared to those who present for PCF. This is consistent with reports from other studies of ACF. However, it is also possible that the number of clinical TB diagnoses does reflect some overdiagnosis.

There is growing evidence that shows that subclinical TB may significantly affect ongoing transmission [25]. Subclinical TB is defined as “disease due to viable M. tuberculosis bacteria that does not cause clinical TB-related symptoms but causes other abnormalities that can be detected using existing radiologic or microbiologic assays” [25]. Importantly, ACF using CXR may aid in detecting such subclinical TB cases, and therefore, could have a role to play in reducing TB transmission. However, questions remain as to optimal contexts, populations, and coverage of ACF to impact TB transmission ACF [8,26].

This study found a high proportion of TB cases, and nearly half of all cases diagnosed by ACF were not reported in the National TB program information system/surveillance in 2021. There were 13 cases in the ACF data with the recorded treatment start date in 2022. Hence, while not reported in the 2021 notification data in SITB, these cases could have been reported in 2022. A further 23 cases could not be accounted for in the SITB data: representing 25% of the 91 cases found through ACF. A high level of unreported cases is consistent with findings from a joint external monitoring mission that identified underreporting as a significant problem in the Indonesian program [27]. Similarly, the Indonesia TB Inventory study from 2016–2017 estimated that 44% of cases diagnosed were unreported, with those clinically diagnosed, extra-pulmonary, and pediatric cases being more likely to be under-reported. Similar to the inventory study results, in our study 23 cases not accounted for in SITB reporting, 87% (n = 20) were clinically diagnosed. Failure to report cases can result in an underestimation of the TB burden in district areas, leading to inadequate program planning and resource allocation. Hence, further investigation is needed to understand why health facilities may not be reporting clinical cases and help district health to review and strengthen TB surveillance in Kulon Progo. 

Limitations of this study include that the number of unreported cases from passive-case findings could not be estimated. Data from SITB may be incomplete or missing and not reflect the actual number of cases diagnosed and started on treatment. There were possibly clinical TB patients diagnosed and treated at primary health care that were not reported in the SITB, and therefore, not identified by this study. The tendency to under-report clinical cases, as observed in the national inventory study, might explain why ACF cases were not reported in SITB. 

Missing or incorrect national identification number (NIK) in SITB and Zero TB Yogyakarta RECDap could have affected linkage and could have resulted in an underestimation of the number of cases from ACF that were actually reported in SITB because linkage could not be made in the absence of the correct NIK in both data sets. If ACF cases were present in the SITB data but could not be linked, this would have resulted in them being counted twice in calculating the total number of cases detected in Kulon Progo in 2021; and would have resulted in misclassification and overestimation of the number of cases that were found in ACF but not reported. Lastly, we could not accurately assess the extent to which ACF resulted in an additional yield of TB cases since there was no comparison with a non-intervention area, and the limitations of longitudinal comparison would have been compounded by the effect of the COVID-19 pandemic. 

## 5. Conclusions

In this rural setting, ACF using chest X-ray detected a higher proportion of clinical TB than passive case finding, and a high proportion of cases detected by ACF were unreported in the notification system. This suggests, that introducing TB ACF programs, attention to reporting of cases, and linkage to cas is important. Improvement of case-finding activities is needed to detect more TB cases, especially those with subclinical TB or who have recently developed active TB that may be missed by existing case-finding systems. 

## Figures and Tables

**Table 1:** The characteristics of TB cases found through active and passive-case finding mechanisms in Kulon Progo in 2021.

Characteristic	PCF, N = 158 ^1^	ACF, N = 91 ^1^	Overall, N = 249 ^1^	*p*-Value ^2^
Age	48 (30, 58)	57 (32, 67)	52 (31, 61)	0.006
Age group				<0.001
0–4 years	10 (6.3%)	3 (3.3%)	13 (5.2%)	
5–14 years	3 (1.9%)	8 (8.8%)	11 (4.4%)	
15–59 years	111 (70.3%)	42 (46.2%)	153 (61.4%)	
60+ years	34 (21.5%)	38 (41.8%)	72 (28.9%)	
Sex				0.4
Female	68 (43.0%)	34 (37.4%)	102 (41.0%)	
Male	90 (57.0%)	57 (62.6%)	147 (59.0%)	
Method of diagnosis				<0.001
Clinically diagnosed	79 (50.0%)	68 (74.7%)	147 (59.0%)	
Bacteriologically confirmed	79 (50.0%)	23 (25.3%)	102 (41.0%)	
Diabetes	11 (17.2%)	10 (24.4%)	21 (20.0%)	0.4
Unknown	94	50	144	
HIV status				>0.9
Negative	38 (95.0%)	11 (100.0%)	49 (96.1%)	
Positive	2 (5.0%)	0 (0.0%)	2 (3.9%)	
Unknown	118	80	198	
Employment status				0.005
Not employed	70 (44.3%)	24 (26.4%)	94 (37.8%)	
Employed	88 (55.7%)	67 (73.6%)	155 (62.2%)	
Provincial zone				0.6
Northern area	35 (22.9%)	19 (21.3%)	54 (22.3%)	
Central area	59 (38.6%)	40 (44.9%)	99 (40.9%)	
Southern area	59 (38.6%)	30 (33.7%)	89 (36.8%)	
Unknown	5	2	7	

^1^ Median (IQR); N (%). ^2^ Wilcoxon rank sum test; Fisher’s exact test; Pearson’s Chi-squared test.

**Table 2:** The characteristic of TB cases reported in Kulon Progo in 2021.

	Summary Statistics				Univariate			
Characteristic	Reported, N = 213 ^1^	Not Reported, N = 23 ^1^	Overall, N = 236 ^1^	*p*-Value ^2^	N	OR ^3^	95% CI ^3^	*p*-Value
Age	52 (31, 60)	50 (19, 65)	52 (30, 61)	0.9	236	0.99	0.97, 1.01	0.5
Age group				0.003	236			
0–4 years	10 (4.7%)	2 (8.7%)	12 (5.1%)			—	—	
5–14 years	6 (2.8%)	4 (17.4%)	10 (4.2%)			3.33	0.49, 29.9	0.2
15–59 years	139 (65.3%)	8 (34.8%)	147 (62.3%)			0.29	0.06, 2.07	0.15
60+ years	58 (27.2%)	9 (39.1%)	67 (28.4%)			0.78	0.17, 5.58	0.8
Sex				0.3	236			
Female	88 (41.3%)	7 (30.4%)	95 (40.3%)			—	—	
Male	125 (58.7%)	16 (69.6%)	141 (59.7%)			1.61	0.66, 4.34	0.3
Method of diagnosis				0.003	236			
Bacteriologically confirmed	97 (45.5%)	3 (13.0%)	100 (42.4%)			—	—	
Clinically diagnosed	116 (54.5%)	20 (87.0%)	136 (57.6%)			5.57	1.84, 24.2	0.007
Employment status				0.016	236			
Not employed	75 (35.2%)	14 (60.9%)	89 (37.7%)			—	—	
Employed	138 (64.8%)	9 (39.1%)	147 (62.3%)			0.35	0.14, 0.83	0.020
Provincial zone				0.8	229			
Northern area	48 (23.1%)	5 (23.8%)	53 (23.1%)			—	—	
Central area	86 (41.3%)	7 (33.3%)	93 (40.6%)			0.78	0.24, 2.76	0.7
Southern area	74 (35.6%)	9 (42.9%)	83 (36.2%)			1.17	0.38, 3.99	0.8
Unknown	5	2	7					

^1^ Median (IQR); N (%). ^2^ Wilcoxon rank sum test; Fisher’s exact test; Pearson’s Chi-squared test. ^3^ OR = Odds Ratio, CI = Confidence Interval.

## Data Availability

The datasets generated and analyzed during the study are available upon request to the corresponding author.
